# Impact of PNPLA3 and IFNL3 polymorphisms on hepatic steatosis in Asian patients with chronic hepatitis C

**DOI:** 10.1371/journal.pone.0182204

**Published:** 2017-08-10

**Authors:** Chao-Min Huang, Kuo-Chin Chang, Chao-Hung Hung, King-Wah Chiu, Sheng-Nan Lu, Jing-Houng Wang, Chien-Hung Chen, Kwong-Ming Kee, Yuan-Hung Kuo, Ming-Chao Tsai, Po-Lin Tseng, Ming-Tsung Lin, Cheng-Kun Wu, Tsung-Hui Hu, Chung-Lung Cho, Yi-Hao Yen

**Affiliations:** 1 Department of Biological Sciences, National Sun Yat-sen University, Kaohsiung, Taiwan; 2 Division of Hepato-Gastroenterology, Department of Internal Medicine, Kaohsiung Chang Gung Memorial Hospital and Chang Gung University College of Medicine, Kaohsiung, Taiwan; Università degli Studi di Palermo, ITALY

## Abstract

**Background and aims:**

A recent meta-analysis revealed that the genotype PNPLA3 rs738409 GG is associated with a higher risk of hepatic steatosis (HS) in Caucasian patients with chronic hepatitis C (CHC). However, controversial results were found regarding Asian populations. Furthermore, previous studies have shown a negative association between interferon lambda 3 (IFNL3) rs12979860 CC and HS in Caucasian CHC patients, but there have been no reports indicating any such association in Asian populations. In this study, then, we investigated the association of PNPLA3 and IFNL3 polymorphisms with HS in Asian CHC patients.

**Methods:**

We enrolled consecutive CHC patients who underwent liver biopsy prior to antiviral therapy. We excluded those patients with decompensated liver disease, any co-existing chronic liver disease, or HIV or HBV co-infection.

**Results:**

1080 CHC patients were enrolled, and HS was found in 453 (41.9%) patients. The frequency distribution of the G allele was significantly associated with HS (P<0.001), and this conferred a higher risk to G allele homozygotes (OR: 2.06, 95% CI: 1.46–2.88, P <0.001) than to G allele carriers (OR: 1.98, 95% CI: 1.52–2.58, P<0.001). There was a borderline significant difference in the prevalence of HS in rs12979860 CC versus non-CC (40.8% versus 49.3%, P = 0.059). After adjustment for age, sex, body mass index, diabetes, and excessive alcohol intake, the rs738409 G allele homozygote carriers still carried a higher risk for HS (OR: 1.93, 95% CI: 1.35–2.77, P = 0.003).

**Conclusion:**

The PNPLA3 rs738409 GG genotype is positively associated with HS, while the IFNL3 rs 12979860 CC genotype may be negatively associated with HS, in Asian CHC patients.

## Introduction

Chronic hepatitis C virus (HCV) infection is one of the most common blood-borne viral infections, affecting more than 170 million individuals worldwide [1–3]. Chronic HCV infection leads to the development of chronic hepatitis, cirrhosis, hepatocellular carcinoma (HCC), and liver-related mortality [4–8].

Hepatic steatosis (HS) is common in patients infected with HCV and appears to be associated with a more rapid progression of liver fibrosis [9–11]. Both host and viral factors, including diabetes, alcohol consumption, older age, higher body mass index (BMI), and HCV genotype 3, are thought to contribute to HCV-related HS [10, 12, 13].

Several genetic risk factors for HS have been identified so far, with the best documented one being a single nucleotide polymorphism (SNP) in rs738409 in the patatin-like phospholipase domain-containing 3 (PNPLA3) gene. This polymorphism is associated with HS in patients with non-genotype-3 chronic hepatitis C (CHC) [14, 15].

A recent meta-analysis revealed that the genotype PNPLA3 rs738409 GG is associated with a higher risk of HS in Caucasian patients with CHC [16]. However, controversial results were found regarding Asian populations [17–20].

Furthermore, previous studies have reported a negative association between the interferon lambda 3 (IFNL3) rs12979860 CC genotype and HS in Caucasian CHC patients [21, 22]. However, there have been no reports indicating any such association in Asian populations.

The aim of this study, therefore, was to investigate the association of PNPLA3 and IFNL3 polymorphisms with HS in Asian CHC patients.

## Methods

A total of 1,080 CHC patients who each received a pre-antiviral evaluation were consecutively recruited at Kaohsiung Chang Gung Memorial Hospital in Kaohsiung City, Taiwan, from 1999 to 2011. All the patients were positive for the anti-HCV antibody (Ax SYM HCV 3.0; Abbott Laboratories, Chicago, IL); qualitative HCV RNA was detected by a PCR-based assay (Cobas Amplicor Hepatitis C Virus Test, version 2.0; Roche Molecular Systems, Branchburg, NJ, USA) with a lower limit of detection of approximately 50 IU/mL. The HCV RNA was quantified by a real-time PCR-based assay (COBAS AmpliPrep/COBAS TaqMan HCV Test; Roche) with a dynamic range of 43–69,000,000 IU/ml, and HCV genotyping was performed using the Siemens Diagnostics Versant HCV Genotype Assay (Munich, Germany).

All of the patients received liver biopsies before initiating antiviral therapy. Excessive alcohol intake was defined as more than 30 grams per day [14]. We excluded those patients with decompensated liver disease, any co-existing chronic liver disease, or HIV or HBV co-infection.

The degree of liver necroinflammation in each patient was calculated by Histology Activity Index scores [23]. The degree of liver fibrosis in each patient was graded and staged according to the modified Knodell histology index [24]. HS was semiquantified according to the four-point scale developed by Brunt [25] by calculating the percentage of lipid-containing hepatocytes among the total number of hepatocytes at a 40x magnification, with one of the following four grades assigned accordingly: grade 0 (<5%), grade 1 (5% to 33%), grade 2 (34 to 66%), and grade 3 (>66%).

All the procedures used in the study were in accordance with the ethical standards of the responsible committees on human experimentation (institutional and national) and with the Helsinki Declaration of 1975, as revised in 2008. This study was approved by the Institutional Review Board of Kaohsiung Chang Gung Memorial Hospital (IRB number: 201601607B0). The requirement for informed consent was waived by the IRB. The data were analyzed anonymously.

### IFNL3 and PNPLA3 genotyping

Genotyping for IFNL3 rs12979860 and rs8099917 and for PNPLA3 rs738409 was carried out using the TaqMan SNP genotyping allelic discrimination method (Applied Biosystems, Foster City, CA, USA). The genotyping was performed with SDS software v.1.3.0 (ABI Prism 7500, Foster City, CA, USA).

### Statistical analysis

The clinical characteristics of the individual patients were summarized as means and standard deviations, frequencies and percentages, or medians and interquartile ranges as appropriate. The distribution of histological characteristics according to the PNPLA3 genotype and steatosis was estimated using the chi-square test, and the relationship between the PNPLA3 genotype, IFNL3 genotype, and HS was determined by univariate logistic regression. Univariate and multivariate logistic regression analyses were used to determine the association of factors associated with histological liver damage, such as HS, necroinflammation, and fibrosis. Post-hoc correction for multiple SNP testing was performed with Bonferroni correction [26]. A p-value of less than 0.05 was considered statistically significant. All analyses were performed by Stata version 11.0.

## Results

### Patients

The mean age of the patients included in the study population was 54.4 years, and 52.2% of the patients were male. Furthermore, 43.9% of the patients were normal weight or underweight, 3.7% of the patients had excessive alcohol intake, and 19.3% of the patients had diabetes mellitus (DM). HS was observed in 453 (41.9%) of the CHC patients. The percentages of patients with grade 0, grade 1, or grade 2 to grade 3 HS were 58.1% (n = 627), 38.8% (n = 419), and 3.1% (n = 34), respectively. Severe necroinflammation (score ≥9) was observed in 391 (36.2%) of the CHC patients, and advanced fibrosis (stage 3 or 4) was observed in 518 (48.0%) of the CHC patients. The PNPLA3 rs738409 CC, CG, and GG genotypes accounted for 34.5%, 50.4%, and 15.1% of the population, respectively. The rs12979860 CC and non-CC genotypes accounted for 87.0% and 13.0% of the population, respectively, while the rs8099917 TT and non-TT genotypes accounted for 87.9% and 12.1% of the population, respectively. The majority of patients were infected with HCV-1 (49.6%) or HCV-2 (45.2%). The genotype could not be identified in 34 (3.2%) patients, while only 6 patients in the current cohort were infected with HCV-3 (Table 1).

**Table 1:** Clinical characteristics of individuals.

Variables	Overall (n = 1080)
Age, years	54.4 ± 10.8
Male	564 (52.2%)
DM	208 (19.3%)
Excessive alcohol intake	37 (3.7%)
BMI (kg/m^2^)	
< 24	473 (43.9%)
24–27	352 (32.7%)
>27	253 (23.5%)
AST (IU/L)	93 (64.5–135)
ALT (IU/L)	130 (95–193)
r-GT (IU/L)	40 (24–78)
Albumin (g/dL)	3.9 ± 0.5
ALK-P (IU/L)	88 (69–115)
Platelet (1000/μL)	16.6 ± 6.0
Viral load >600,000 IU/mL	188 (20.9%)
Genotype	
1	536 (49.6%)
2	488 (45.2%)
others	22 (2.0%)
unknown	34 (3.2%)
Necroinflammation score	
<9	689 (63.8%)
≥9	391 (36.2%)
Fibrosis stage	
0–2	562 (52.0%)
3–4	518 (48.0%)
Steatosis	
<5%	627 (58.1%)
5%-33%	419 (38.8%)
>33%	34 (3.1%)
PNPLA3	
GG	163 (15.1%)
non-GG	917 (84.9%)
rs12979860	
CC	940 (87.0%)
non-CC	140 (13.0%)
rs8099917	
TT	949 (87.9%)
non-TT	131 (12.1%)

Data were expressed as mean ± SD or median (interquantile). DM, diabetes mellitus; BMI, body mass index; AST, Aspartate Aminotransferase; ALT, Alanine Aminotransferase; r-GT, r-Glutamyl Transpeptidase; ALK-P, Alkaline phosphatase PNPLA3, patatin-like phospholipase domain-containing 3.

### Association between the IFNL3 polymorphism and HS

There was a borderline significant difference in the prevalence of HS in the rs12979860 CC genotype versus the rs12979860 non-CC genotype (40.8% versus 49.3%, P = 0.059). There was also a borderline significant difference in the prevalence of HS in the rs8099917 TT genotype versus the rs8099917 non-TT genotype (40.9% versus 49.6%, P = 0.058).

### Interaction between the IFNL3 and PNPLA3 genotypes in the pathogenesis of HS

The rs12979860 CC genotype provided protection from HS in patients who were positive, but not in those who were negative, for the PNPLA3 G variant (281/707, 46.0% vs. 55/96, 57.3%, P = 0.039 and 103/329, 31.3% vs. 14/44, 31.8%, P = 0.945, respectively). The rs809917 TT genotype provided protection from HS in patients who were positive, but not in those who were negative, for the PNPLA3 G variant (284/616, 46.1% vs. 52/91, 57.1%, P = 0.049 and 103/333, 31.2% vs. 13/40, 32.5%, P = 0.870, respectively) (Table 2).

**Table 2:** Interaction between the IFNL3 and PNPLA3 genotypes in the pathogenesis of steatosis.

	rs12979860 CC	rs12979860 non-CC	P value	rs8099917 TT	rs8099917 non-TT	P value
PNPLA3 GG+CG and steatosis ≥5%	281 (46.0%)	55 (57.3%)	0.039	284 (46.1%)	52 (57.1%)	0.049
PNPLA3 CC and steatosis ≥5%	103 (31.3%)	14 (31.8%)	0.945	104 (31.2%)	13 (32.5%)	0.870

PNPLA3, patatin-like phospholipase domain-containing 3; IFNL3, interferon lambda 3.

### Association between the PNPLA3 polymorphism and histological damage

The frequency distribution of the G allele was significantly associated with HS (P<0.001), and this conferred a higher risk to G allele homozygotes (odds ratio (OR): 2.06, 95% confidence interval (CI): 1.46–2.88, P <0.001) than to G allele carriers (OR: 1.98, 95% CI: 1.52–2.58, P<0.001). However, the frequency of the rs738409 G allele was not significantly higher in patients with advanced fibrosis (F3-F4 stages) than in patients at less advanced stages (F0-F2 stages; P = 0.086). The frequency of the rs738409 G allele was not significantly higher in patients with severe necroinflammation (necroinflammation score ≥9) than in patients with less severe necroinflammation (necroinflammation score <9; P = 0.864) (Table 3).

**Table 3:** Histological characteristics according to PNPLA3 (rs738409 C>G) genotype and related odds ratios.

	Genotype				
	CC		CG	GG	P value
Steatosis					<0.001
<5%	256 (68.6%)		301 (55.3%)	70 (42.9%)	
≥5%	117 (31.4%)		243 (44.7%)	93 (57.1%)	
Fibrosis stage					0.086
0–2	192 (51.5%)		297 (54.6%)	73 (44.8%)	
3–4	181 (48.5%)		247 (45.4%)	90 (55.2%)	
Necroinflammation score					0.864
<9	240 (64.3%)		348 (64.0%)	101 (62.0%)	
≥9	133 (35.7%)		196 (36.0%)	62 (38.0%)	
	Dominant Model (CG + GG Versus CC Genotypes)			Recessive Model (GG Versus CG + CC Genotypes)	
	OR (95% CI)	P Value		OR (95% CI)	P Value
Steatosis≥5%	1.98 (1.52–2.58)	<0.001		2.06 (1.46–2.88)	<0.001
Fibrosis stage 3–4	0.97 (0.75–1.24)	0.788		1.41 (1.01–1.97)	0.045
Necroinflammation score≥9	1.04 (0.80–1.35)	0.786		1.10 (0.78–1.55)	0.597

Abbreviations: CI, confidence interval; OR, odds ratio; PNPLA3, patatin-like phospholipase domain-containing 3.

### Logistic regression model for histological damage

In light of the above results indicating a greater influence of rs738409 in G allele homozygotes, we conducted univariate and multivariate logistic regression analyses for histological damage that included the rs738409 genotype using a recessive model (GG genotype versus GC+CC genotypes) as a reference and also included the reported clinical factors (Table 4). After adjustment, G allele homozygotes remained independently associated with HS. In addition, BMI was an independent predictor of HS (P<0.001). However, PNPLA3 G allele homozygotes and the IFNL3 favorable genotype were not associated with advanced fibrosis and severe necroinflammation.

**Table 4:** Univariate and multivariable logistic regression analysis of PNPLA3 and IFNL3 influence on histological liver damage characteristics.

	Univariate			Multivariate		
Variables	OR	95%CI	*P*	OR	95%CI	P*
Steatosis ≥5% (N = 453)						
PNPLA3 GG vs GC+CC	2.06	1.47–2.88	<0.001	1.93	1.35–2.77	0.003
Age (years)	1.00	0.99–1.01	0.637	1.01	0.99–1.02	1.000
Male	0.92	0.72–1.17	0.492	0.89	0.68–1.15	1.000
BMI (kg/m^2^)	1.12	1.08–1.16	<0.001	1.13	1.09–1.17	<0.001
DM	1.47	1.08–1.99	0.013	1.33	0.96–1.84	0.765
Excessive alcohol intake	0.66	0.33–1.32	0.238	0.80	0.39–1.65	1.000
rs12979860 CC	0.71	0.50–1.01	0.060	0.70	0.19–2.62	1.000
rs8099917 TT	0.70	0.49–1.01	0.058	1.12	0.29–4.33	1.000
Fibrosis stage 3–4 (N = 518)						
PNPLA3 GG vs GC+CC	1.41	1.01–1.97	0.045	1.41	0.98–2.02	0.564
Age (years)	1.05	1.03–1.06	<0.001	1.05	1.03–1.06	<0.001
Male	0.78	0.62–0.99	0.044	0.87	0.67–1.13	1.000
BMI (kg/m^2^)	1.03	0.99–1.06	0.095	1.03	1.00–1.07	0.590
DM	1.89	1.39–2.57	<0.001	1.55	1.12–2.16	0.074
Excessive alcohol intake	0.98	0.51–1.89	0.950	1.47	0.73–2.94	1.000
rs12979860 CC	0.65	0.46–0.94	0.020	1.27	0.34–4.74	1.000
rs8099917 TT	0.63	0.44–0.91	0.015	0.56	0.15–2.18	1.000
Necroinflammation score ≥9						
(N = 391)						
PNPLA3 GG vs GC+CC	1.10	0.78–1.55	0.597	1.12	0.78–1.60	1.000
Age (years)	1.02	1.01–1.04	<0.001	1.02	1.01–1.03	0.032
Male	0.70	0.55–0.90	0.005	0.71	0.54–0.92	0.098
BMI (kg/m^2^)	1.00	0.96–1.03	0.826	1.00	0.96–1.04	1.000
DM	1.37	1.01–1.87	0.043	1.28	0.92–1.77	1.000
Excessive alcohol intake	0.93	0.47–1.84	0.829	1.25	0.61–2.53	1.000
rs12979860 CC	1.02	0.71–1.48	0.897	1.52	0.37–6.21	1.000
rs8099917 TT	1.02	0.69–1.49	0.934	0.71	0.17–3.00	1.000

P*: Post-hoc correction for multiple testing was performed with Bonferroni correction. Abbreviations: CI, confidence interval; OR, odds ratio; BMI, body mass index; DM, diabetes mellitus; PNPLA3, patatin-like phospholipase domain-containing 3.

### Role of IFNL3 and PNPLA3 SNPs in determining HS among patients with different BMIs

Because BMI, IFNL3 SNPs, and PNPLA3 SNPs are important determinants of HS, we further analyzed the influence of these SNPs in HS among patients with different BMIs. Patients were categorized as normal weight or underweight (<24 kg/m^2^), overweight (24–27 kg/m^2^), or obese (>27 kg/m^2^) according to the definition of the Health Promotion Administration of the Ministry of Health and Welfare in Taiwan [27]. Among the normal or underweight patients, HS was associated with older age, a higher proportion of patients with DM, the PNPLA3 GG genotype, and a lower proportion of patients with the IFNL3 favorable genotype in the univariate analysis (Table 5). In the multivariate analysis, no factor was associated with HS (Table 6). However, the PNPLA3 GG genotype was the only factor associated with HS (OR: 1.89, 95% CI: 1.12–3.20, P = 0.018) if the variables of IFNL3 SNPs were not taken into consideration (Table 7). Among the overweight patients, HS was associated with a lower proportion of male patients and a higher proportion of patients with the PNPLA3 GG genotype. In multivariate analysis, no factor was associated with HS (Table 6). However, the PNPLA3 GG genotype was the only factor associated with HS (OR: 2.37, 95% CI: 1.25–4.47, P = 0.008) if the variables of IFNL3 SNPs were not taken into consideration (Table 7). For obese patients, HS was associated with a higher proportion of patients with PNPLA3 GG+GC genotype. In multivariate analyses with or without IFNL3 SNPs taken into consideration, no factor was associated with HS (Tables 6 and 7).

**Table 5:** Univariate analysis of factors associated with hepatic steatosis stratified by body mass index.

Variables	BMI<24			BMI 24–27			BMI>27		
	Steatosis (-) (N = 315)	Steatosis (+) (N = 158)	*P*	Steatosis (-) (N = 196)	Steatosis (+) (N = 156)	*P*	Steatosis (-) (N = 115)	Steatosis (+) (N = 138)	*P*
Age, years	54.0 ± 11.8	56.1 ± 9.6	0.048	54.7 ± 9.8	54.9 ± 10.2	0.849	53.9 ± 11.9	52.3 ± 10.5	0.262
Male	145 (46.0%)	79 (50.0%)	0.415	128 (62.3%)	83 (53.2%)	0.021	59 (51.3%)	68 (49.3%)	0.748
DM	43 (13.7%)	33 (21.0%)	0.040	30 (15.3%)	36 (23.1%)	0.064	32 (27.8%)	34 (24.7%)	0.565
Platelet (1000/μL)	12.9 ± 6.1	16.0 ± 5.1	0.115	16.6 ± 6.3	16.1 ± 5.6	0.430	16.1 ± 6.5	17.9 ± 6.7	0.030
AST (IU/L)	94 (65–129)	98 (71–142)	0.304	87 (59–135)	102.5 (71–144)	0.422	91.5 (62–143)	86 (65–123)	0.356
ALT (IU/L)	123 (91–182)	139 (99–203)	0.185	122 (87–200)	147 (99–213)	0.422	121 (91–192)	134 (98–193)	0.742
Genotype 1	150 (49.0%)	68 (45.3%)	0.077	112 (60.5%)	67 (43.5%)	0.004	68 (59.7%)	70 (51.9%)	0.302
Viral load >600,000 IU/mL	44 (16.6%)	26 (20.3%)	0.359	36 (21.6%)	24 (18.1%)	0.450	30 (29.4%)	28 (26.9%)	0.691
Fibrosis stage 3–4	136 (43.2%)	75 (47.5%)	0.376	100 (51.0%)	85 (54.5%)	0.518	61 (53.0%)	60 (43.5%)	0.129
Necroinflammation score ≥9	107 (34.0%)	70 (44.3%)	0.028	55 (28.1%)	71 (45.5%)	0.001	49 (42.6%)	38 (27.5%)	0.012
PNPLA3 GG	39 (12.4%)	37 (23.4%)	0.002	18 (9.2%)	34 (21.8%)	0.001	13 (11.3%)	22 (15.9%)	0.287
PNPLA3 GG+GC	192 (61.0%)	123 (77.9%)	<0.001	114 (58.2%)	114 (73.1%)	0.004	65 (56.5%)	99 (71.7%)	0.012
Excessive alcohol intake	11 (3.7%)	5 (3.5%)	0.913	12 (6.9%)	5 (6.4%)	0.168	2 (1.9%)	2 (1.6%)	1.000
rs12979860CC	282 (89.5%)	128 (81.0%)	0.010	171 (87.2%)	132 (84.6%)	0.479	102 (88.7%)	123 (89.1%)	0.913
rs8099917 TT	284 (90.2%)	129 (81.7%)	0.009	174 (88.8%)	134 (85.9%)	0.417	102 (88.7%)	124 (89.8%)	0.766

Data were expressed as mean ± SD or median (interquantile). Abbreviations: DM, diabetes mellitus; BMI, body mass index; AST, Aspartate Aminotransferase; ALT, Alanine Aminotransferase; PNPLA3, patatin-like phospholipase domain-containing 3.

**Table 6:** Multivariable logistic regression analysis of factors associated with hepatic steatosis stratified by body mass index.

BMI (kg/m^2^)	Variables	OR	95%CI	P*
<24 (n = 473)				
	PNPLA3 GG vs GC+CC	1.92	1.13–3.26	0.131
	Age (years)	1.02	1.00–1.04	0.948
	Male	1.03	0.68–1.58	1.000
	DM	1.58	0.92–2.70	0.776
	Excessive alcohol intake	1.10	0.35–3.39	1.000
	rs12979860 CC	0.80	0.12–5.32	1.000
	rs8099917 TT	0.61	0.09–4.24	1.000
BMI 24–27 (n = 352)				
	PNPLA3 GG vs GC+CC	2.42	1.27–4.63	0.059
	Age (years)	1.00	0.97–1.02	1.000
	Male	0.64	0.40–1.03	0.514
	DM	1.67	0.93–2.98	0.674
	Excessive alcohol intake	0.60	0.20–1.78	1.000
	rs12979860 CC	0.70	0.10–5.13	1.000
	rs8099917 TT	1.58	0.20–12.7	1.000
BMI>27 (n = 253)				
	PNPLA3 GG vs GC+CC	1.46	0.67–3.15	1.000
	Age (years)	0.99	0.97–1.02	1.000
	Male	0.86	0.51–1.45	1.000
	DM	0.88	0.49–1.57	1.000
	Excessive alcohol intake	0.75	0.10–5.71	1.000
	rs12979860 CC	1.11	0.48–2.56	1.000

P*: Post-hoc correction for multiple testing was performed with Bonferroni correction. Abbreviations: CI, confidence interval; OR, odds ratio; DM, diabetes mellitus; BMI, body mass index; PNPLA3, patatin-like phospholipase domain-containing 3.

**Table 7:** Multivariable logistic regression analysis of factors associated with hepatic steatosis stratified by body mass index without IFNL3 SNPs taken into consideration.

BMI (kg/m^2^)	Variables	OR	95%CI	*P*
<24 (n = 473)				
	PNPLA3 GG vs GC+CC	1.89	1.12–3.20	0.018
	Age (years)	1.02	1.00–1.04	0.094
	Male	1.03	0.68–1.57	0.887
	DM	1.67	0.99–2.84	0.056
	Excessive alcohol intake	1.13	0.37–3.43	0.834
BMI 24–27 (n = 352)				
	PNPLA3 GG vs GC+CC	2.37	1.25–4.47	0.008
	Age (years)	1.00	0.97–1.02	0.853
	Male	0.64	0.40–1.02	0.063
	DM	1.64	0.92–2.93	0.091
	Excessive alcohol intake	0.59	0.20–1.75	0.337
BMI>27 (n = 253)				
	PNPLA3 GG vs GC+CC	1.47	0.68–3.16	0.331
	Age (years)	0.99	0.97–1.02	0.688
	Male	0.86	0.51–1.46	0.582
	DM	0.88	0.49–1.57	0.666
	Excessive alcohol intake	0.76	0.10–5.75	0.787

Abbreviations: CI, confidence interval; OR, odds ratio; DM, diabetes mellitus; BMI, body mass index; PNPLA3, patatin-like phospholipase domain-containing 3.

## Discussion

Approximately 40% of the CHC patients in the study cohort had HS, a rate which was comparable to the prevalence rates reported by studies conducted in Western countries [28–30].

A recent meta-analysis revealed that the genotype PNPLA3 rs738409 GG is associated with a higher risk of HS in Caucasian patients with CHC [16]. However, controversial results were found regarding Asian populations [17–20].

In the current large-scale study, we demonstrated that the influence of the PNPLA3 genetic variants in HS remains consistent in Asian CHC populations. The association was particularly enhanced through the use of the recessive model. CHC patients who carried the PNPLA3 rs738409 GG genotype had a 2.06-fold risk of developing HS when compared to their counterparts.

Both PNPLA3 genetic variants and BMI played important roles in HS in CHC patients. Importantly, we identified that the host genetic effect was mainly restricted to non-obese patients (BMI ≤ 27 kg/m^2^) and not to obese patients (BMI>27 kg/m^2^). For patients with normal or under weight (BMI < 24 kg/m^2^), the carriage of the PNPLA3 rs738409 GG genotype increased the risk of HS 1.89-fold when compared to those individuals carrying the GC+CC genotype. For patients with overweight (BMI 24–27 kg/m^2^), the carriage of the PNPLA3 rs738409 GG genotype increased the risk of HS 2.37-fold when compared to those individuals carrying the GC+CC genotype.

However, the PNPLA3 rs738409 GG genotype was not associated with advanced fibrosis and severe necroinflammation in our study. This was in contrast, to some extent, with a recent meta-analysis that revealed, through a subgroup analysis by ethnicity, that in the Caucasian population, CHC patients with the GG genotype had a higher risk of advanced fibrosis compared with those with genotype CC+CG (OR: 2.51, 95% CI: 1.75–3.60, P<0.05). However, this association was not observed in the Asian population (OR: 1.31, 95% CI: 0.65–2.67, P = 0.457), in spite of the fact that there were no statistically significant differences between the two populations in terms of BMI, age, or gender ratio [16]. The authors [16] explained that this result may have been related to the IFNL3 gene polymorphism, which is another factor associated with the severity of liver disease in CHC [31].

A previous study showed that the PNPLA3 GG genotype was not associated with the risk of developing severe liver necroinflammation [32], a finding which is consistent with the results of the present study.

HCV by itself, especially genotype 3, may lead to HS, but the determination of the effects of the PNPLA3 polymorphism on HS has been restricted to non-genotype 3 chronic HCV-infected patients [33, 34]. In our study, only 6 patients were genotype 3.

HS develops in about 90% of individuals who have excessive alcohol intake [35]. However, excessive alcohol intake (defined as more than 30 grams per day) was not associated with HS in our study. The same finding was noted in a previous study [14].

The PNPLA3 rs738409 variant has now been associated with HS in cases of chronic liver diseases with different etiologies, including alcoholic liver disease [36], CHC [16], and non-alcoholic fatty liver disease [37]. Furthermore, this association appears to be consistent among patients of different ethnicities. As such, an explication of the physiological functions and pathological effects of PNPLA3 may lead to the identification of therapeutic targets for these diseases.

Previous studies have reported a negative association between the IFNL3 rs12979860 CC genotype and HS in Caucasian CHC patients [21, 22]. Also, the IFNL3 rs12979860 CC genotype has been found to be strongly associated with sustained virological response (SVR) in patients who undergo pegylated interferon plus ribavirin combination therapy [38–40], while HS and insulin resistance have also been demonstrated to be associated with SVR [41, 42]. The IFNL3 genotype is associated with both insulin resistance and HS [43], which could partly explain the associations among the IFNL3 genotype, HS, and SVR.

In East Asian populations, including that of Taiwan, the favorable IFNL3 genotype is very common [44–46]. In the present study, in which nearly 90% of the study population had the favorable IFNL3 genotype, there were still negative associations of IFNL3 rs12979860 CC and rs8099917 TT with HS, although the relevant P values indicated that the significance of these associations was borderline. Further studies with larger sample sizes, however, may be able to clarify the association between the IFNL3 genotype and HS in East Asian populations.

Previous studies also showed that the IFNL3 genotype was not associated with the risk of developing advanced liver fibrosis or severe necroinflammation [47, 48], a finding which is likewise consistent with the results of the present study. However, other studies showed that the IFNL3 genotype were associated with the risk of developing advanced liver fibrosis or severe necroinflammation [49–54]. The discrepancy between studies could be attributed to different sample size, ethnic variation and hence Minor allele frequency. Our study enrolled 1080 Asian CHC patients. In contrast, other studies enrolled 1483~3234 Caucasian CHC patients [49–54]. Eighty seven % of patients had rs12979860CC genotype in our study. In contrast, 34~57% of patients had rs12979860CC genotype in other studies [49–54].

A previous study suggested the possibility that an interaction occurs between the IFNL3 and PNPLA3 genotypes in the pathogenesis of HS in non-genotype 3 chronic HCV-infected patients. The rs12979860 CC genotype was found to provide protection from HS in those who were positive, but not in those were negative, for the PNPLA3 G variant [55]. The same finding was also noted in the present study, in which both the rs12979860 CC genotype and the rs8099917 TT genotype were found to provide protection from HS in those who were positive, but not in those who were negative, for the PNPLA3 G variant.

The strength of the present study is its large sample size and the biopsy-proven grading of HS. The limitations of this study consisted of the retrospective analysis and the absence of metabolic profiles, such as lipid profile and hypertension, both of which may have interfered with the final results.

In conclusion, we demonstrated that the PNPLA3 GG genotype is positively associated with HS, while the favorable IFNL3 genotype may be negatively associated with HS, in Asian CHC patients. In addition, we found that the rs12979860 CC genotype and the rs8099917 TT genotype provide protection from HS in patients who are positive, but not in those who are negative, for the PNPLA3 G variant.

## Supporting information

S1 Dataset The raw data of the patients. (XLSX)
